# Upregulation of genes encoding digestive enzymes and nutrient transporters in the digestive system of broiler chickens by dietary supplementation of fiber and inclusion of coarse particle size corn

**DOI:** 10.1186/s12864-018-4592-2

**Published:** 2018-03-20

**Authors:** Sarbast K. Kheravii, Robert A. Swick, Mingan Choct, Shu-Biao Wu

**Affiliations:** 1 Animal Science, School of Environmental and Rural Science, University of New England, Elm Avenue, Armidale, NSW 2351 Australia; 2 Animal production, Faculty of Agriculture and Forestry, University of Duhok, Duhok, Kurdistan 42003 Iraq

**Keywords:** Gene expression, Sugarcane bagasse, Fiber, Particle size, Amylase, Pepsinogen, Nutrient transporter, Broilers

## Abstract

**Background:**

Measures to improve bird performance have been sought due to the imminent phase out of in-feed antibiotics in poultry and continued demand for higher poultry feeding efficiency. Increasing grain particle size and dietary fibre may improve gizzard function, digestive efficiency and nutrient absorption. This study was conducted to evaluate the effect increased particle size of corn and inclusion of sugarcane bagasse (SB) on mRNA expression of genes encoding digestive enzymes and nutrient transporters in broilers.

**Results:**

A total of 336 day-old Ross 308 males were assigned in a 2 × 2 factorial arrangement of treatments with corn particle size - coarse 3576 μm or fine 1113 μm geometric mean diameter, and SB - 0 or 2% inclusion. Feed conversion ratio (FCR), weight gain and feed intake were measured from d 0–10 and d 10–24. The relative gizzard weight and mRNA expression of genes encoding digestive enzymes and intestinal nutrient transporters were measured on d 24. During d 10–24, a particle size × SB interaction was observed for FCR (*P* < 0.01), where birds fed coarsely ground corn (CC) with 2% SB had lower FCR than those fed CC without SB. A particle size × SB interaction was observed for both expression of pepsinogen A and C (P < 0.01) which were negatively correlated with FCR on d 24. Addition of 2% SB upregulated pepsinogen A and C only in CC fed birds. Further, 2% SB also upregulated pancreatic amylase (AMY2A) and intestinal cationic amino acid transporter-1 (CAT1). Inclusion of dietary CC upregulated duodenal amino peptidase N (APN), jejunal alanine, serine, cysteine and threonine transporter-1 (ASCT1), and ileal peptide transporter-2 (PepT2).

**Conclusion:**

These results suggest that both SB and coarse particle size modulate expression of genes encoding important digestive enzymes and nutrient transporters and thus are directly related to bird performance. These findings provide insights into the combination effects of dietary fiber and particle size in the future management of broiler feeding.

## Background

Consumer pressure and legislation to ban the use of in-feed antibiotics in the EU and voluntarily removal of antibiotics from animal feed in other countries have supressed performance and profitability in broiler chickens due to enteric disorders [1, 2]. The development of antibiotic alternatives to improve digestive efficiency is becoming a topic of broad interest for the poultry industry worldwide. Strategies to improve broiler digestive efficiency and performance without reliance on antibiotics have been the focus for improving gut health and manipulating development of the gastrointestinal tract (GIT). A range of nutritional interventions including; increasing grain particle size, using whole grain, and including different sources or levels of dietary fiber and other feed additives, such as probiotics, bacteriophages, enzymes and phytobiotics are currently under investigation [2–4].

The physical structure of feed ingredients and inclusion of dietary fiber may improve nutrient digestion and absorption as a result of increased gizzard size [3, 5] and enhanced secretion of HCl in the proventriculus [6]. Low pH in the upper GIT is known to improve solubility and absorption of minerals [7] and increase pepsin activity [8]. Well-developed gizzard musculature has been hypothesized to elevate pancreatic enzyme secretion including amylase and chymotrypsin through increased release of cholecystokinin [9]. The inclusion of fiber or coarse particle size in broiler diets not only enhances gizzard development but also increases digesta retention time and gut reflux [9, 10]. Slower digesta retention time improves nutrient digestion and absorption by increasing time of contact with absorptive cells [11]. In the chickens, the small intestine is the main site of nutrient absorption. It is known that the glucose transporters: glucose transporter-1 (GLUT1) and glucose transporter 2 (GLUT2); amino acid transporters: Na + −dependent neutral amino acid transporters, such as B^o^AT and ASCT1, cationic amino acid transporters, such as CAT1 and CAT2, and Na + −dependent neutral/cationic amino acid exchanger, such as y^+^ L amino acid transporter-1 and y^+^ L amino acid transporter-2, and peptide transporters, such as PepT1 and PepT2, in the small intestinal epithelium are closely associated with nutrient absorption capacity [12, 13]. Although, structural components of the diet have been reported to improve nutrient digestibility and performance in broilers [3, 14–17], there are no investigations on nutrigenomic mechanisms underlying such improvements.

This study investigated the influence of fiber supplementation and increased corn particle size on broiler performance at the gene expression level. It was hypothesized that fiber and coarse particle size would stimulate secretion of digestive enzymes and nutrient transporters that would then accelerate the digestive activity in the intestine and improve feed conversion efficiency.

## Methods

### Experimental design, bird management and diet

A total of 336 d-old male Ross 308 chicks were obtained on the hatching day from a local hatchery (Baiada Hatchery in Tamworth, NSW, Australia). The chicks were assigned in a 2 × 2 factorial arrangement of treatments with 2 particle sizes (coarsely ground corn, CC, 3576 μm or finely ground corn, FC, 1113 μm geometric mean diameter, GMD) and 2 levels of sugarcane bagasse (0%, 2%). The geometric mean diameter of corn particle size was determined according to the American Society of Agricultural Engineers (2003). The birds were randomly allocated to 4 treatments with 6 replicate pens each stocked with 14 birds. The broiler chicks were reared in pens measuring 75 cm × 120 cm to 24 d. Hardwood shavings were used as bedding with an initial depth of 7 cm. Each pen was equipped with a single tube feeder and 2 nipple drinkers. Feed and water were provided ad libitum. The lighting, relative humidity and temperature followed Ross 308 strain guidelines [18].

Table 1 shows the ingredient and nutrient composition of experimental diets. The diets were formulated to Ross 308 specifications [18]. The composition of starter and grower diets was diluted when 2% SB added over the top of the complete feed. All diets were thoroughly mixed and cold-pelleted (65 °C). The feeding program consisted of a starter (d 0 to 10), and grower (d 11 to 24).

**Table 1:** Composition and nutrient content of corn base diet (%)

Ingredients	Starter	Grower
Corn	60.6	62.3
Soybean meal	32.6	29.3
Meat and bone meal	3.00	3.60
Canola oil	0.644	1.91
Limestone	0.970	0.814
Dical phosphate	0.607	0.269
Phytase^a^	0.01	0.010
Salt	0.154	0.161
Na bicarbonate	0.219	0.200
Vitamin premix^b^	0.200	0.200
Choline	0.111	0.103
L-lysine HCl 784	0.305	0.226
D, L-methionine	0.392	0.336
L-threonine	0.204	0.148
TiO_2_	–	0.500
Nutrients		
ME (kcal/kg)	3000	3100
ME (MJ/kg)	12.55	12.97
Crude protein	22.2	21.0
Crude fat	2.85	4.14
Crude Fiber	2.07	2.01
SID arginine	1.37	1.27
SID lysine	1.28	1.15
SID methionine	0.684	0.616
SID methionine + Cysteine	0.950	0.870
SID tryptophan	0.244	0.226
SID isoleucine	0.860	0.807
SID threonine	0.860	0.770
SID valine	0.992	0.939
Starch	35.8	36.8
NSP soluble	0.426	0.404
NSP insoluble	5.64	5.45
Calcium	0.960	0.870
Available Phosphorus	0.480	0.435
Sodium	0.160	0.160
Chloride	0.250	0.242
Choline	0.170	0.160

^a^Phyzyme XP5000G (100 g/mt) Dupont

^b^Vitamin-Mineral concentrate supplied per kilogram of diet: retinol, 12,000 IU; cholecalciferol, 5000 IU; tocopheryl acetate, 75 mg, menadione, 3 mg; thiamine, 3 mg; riboflavin, 8 mg; niacin, 55 mg; pantothenate, 13 mg; pyridoxine, 5 mg; folate, 2 mg; cyanocobalamine, 16 μg; biotin, 200 μg; cereal-based carrier, 149 mg; mineral oil, 2.5 mg; Cu (sulphate), 16 mg; Fe (sulphate), 40 mg; I (iodide), 1.25 mg; Se (selenate), 0.3 mg; Mn (sulphate and oxide), 120 mg; Zn (sulphate and oxide), 100 mg; cereal-based carrier, 128 mg; mineral oil, 3.75 mg; SID = Standard ileal digestible

The SB was provided by FCR Consulting Group, Brisbane. The composition of SB was determined (“as is” basis) for total non-starch polysaccharides (NSP) and lignin following the method described by Englyst et al. [19] and Kirk and Obst [20], respectively. The SB contained 6.1 g/kg free sugar, 191 g/kg lignin, 534 g/kg insoluble NSP and 1.9 g/kg soluble NSP.

### Measurement of growth performance and gizzard weight

Birds and leftover feed in each pen (*n* = 6/treatment) were weighed and the average weight gain, feed intake, and FCR were calculated based on the measurements at the end of the starter, d 10, and grower, d 24 phases. On d 24, empty gizzards without proventriculi from 3 birds per pen were weighed and recorded along with corresponding bird weights. The relative gizzard weight was calculated as mass per unit of live body weight (g/100 g of live body weight).

### Sampling and RNA isolation

On d 24, one bird was randomly selected from each pen and killed by cervical vertebrae dislocation. Around 2 cm from each duodenum, jejunum, ileum, proventriculus and pancreas was excised and flushed with 4 °C PBS and collected into a 2 mL Eppendorf cap lock tube, snap-frozen in liquid N_2_, and kept at − 80 °C until required for RNA extraction. For each sample, total RNA was extracted from the tissue after homogenization in TRIsure™ (Bioline, Sydney, Australia) following the manufacturer’s instructions. Total RNA quantity and purity was determined using a NanoDrop ND-8000 spectrophotometer (Thermo Fisher Scientific, Waltham, USA). An RNA 6000 Nano kit was used to measure RNA integrity (RNA Integrity Number, or RIN) using the Agilent 2100 Bioanalyzer (Agilent Technologies, Inc., Waldbronn, Germany). The RNA samples were considered of high integrity if the RIN was higher than 7.5 [21]. The RIN values of the samples were 7.9–9.8 in the present study.

### cDNA synthesis

The isolated RNA of each sample was reverse-transcribed with the QuantiTect Reverse Transcription Kit (Qiagen, Hilden, Germany) following manufacturer’s instructions (gDNA elimination step performed). The Rotorgene 6000 real-time PCR machine (Corbett, Sydney, Australia) was employed to convert the RNA into cDNA. The cDNA was diluted three times with nuclease-free water and stored at − 20 °C until required.

### Primer sources and design

In the current study, the primers were either sourced from previously published studies in chickens or designed using NCBI primer tool (https://www.ncbi.nlm.nih.gov/). Table 2 show the primers that were used in this study. Prior to qPCR analysis, Agilent 2100 Bioanalyzer (Agilent Technologies, Inc., Germany) was employed to check the primer specificity for each pair using Agilent DNA 1000 Kit (Agilent Technologies, Inc., Germany). Only primer pairs with specific amplifications were used in this study.

**Table 2:** Sequences of primers used for quantitative real-time PCR

Gene	Gene full name	Primer sequence (5′-3′)	Exons spanning	Ta	size (bp)	Reference	Accession No.
ATP1A1	ATPase Na+/K+ transporting subunit alpha 1	F-GTCAACCCGAG^↓^GGATGCTAA R-ACTGCTACAATGGCACCCTG	15/16 N/A	60	179	This study	NM_205521.1
AMY2A	Pancreatic alpha 2A amylase	F-CGGAGTG^↓^GATGTTAACGACTGG R-ATGTTCGCAGACCCAGTCATTG	7/8 N/A	60	112	This study	NM_001001473.2
APN	Aminopeptidase N	F-AATACGCGCTCGAGAAAACC R-AGCGGGTACGCCGTGTT	N/A N/A	60	70	[40]	NM_204861.1
ASCT1	Alanine, serine, cysteine, and threonine transporter (SLC1A4)	F-TTGGCCGGGAAGGAGAAG R-AGACCATAGTTGCCTCATTGAATG	N/A N/A	60	63	[41]	XM_001232899.4
B^0^AT	Solute carrier family 6, member 19 (SLC6A19)	F-GTGTTTGGAACCCTAAATAC^↓^GAGG R-TAGCATAGACCCAGCCAGGA	12/13 N/A	60	72	This study	XM_419056.5
b^o,+^AT	Solute carrier family 7, member 9 (SLC7A9)	F-CAGTAGTGAATTCTCTGAGTGTGAAGCT R- GCAATGATTGCCACAACTACCA	N/A N/A	60	88	[40]	NM_001199133.1
CAT1	Cationic amino acid transporter-1 (SLC7A1)	F-CAAGAGGAAAACTCCAGTAATTGCA R- AAGTCGAAGAGGAAGGCCATAA	N/A N/A	60	75	[40]	XM_015277945.1
CAT2	Cationic amino acid transporter-2 (SLC7A2)	F-TGCTCGCGTTCCCAAGA R- GGCCCACAGTTCACCAACAG	N/A N/A	60	67	[40]	XM_015285435.1
CCK1R	Cholecystokinin type 1 receptor	F-CACTTACTTCATGGGTATCTCTGTG R-GATGGCAACAAGGTTGAATGTAGA	N/A N/A	60	55	[42]	AB214534.1
CCK	Cholecystokinin	F-AGGTTCCACTGGGAGGTTCT R-CGCCTGCTGTTCTTTAGGAG	N/A N/A	60	152	This study	XM_015281332.1
CELA1	Chymotrypsin-like elastase family, member 1	F-AGCGTAAGGAAATGGGGTGG R-GTGGAGACCCCATGCAAGTC	N/A N/A	60	75	This study	XM_015300368.1
CELA2A	Chymotrypsin like elastase family member 2A	F-GAGGGGAAGATGCAAGACCAT R-CCTTGCTCCTCAGCTTCTAGG	N/A N/A	60	196	This study	NM_001032390.2
EAAT3	Excitatory amino acid transporter 3 (SLC1A1)	F-TGCTGCTTTGGATTCCAGTGT R-AGCAATGACTGTAGTGCAGAAGTAATATATG	N/A N/A	60	79	[43]	XM_424930.5
GLUT1	Glucose transporter-1 (SLC2A1)	F-TCCTCCTGATCAACCGCAAT R-TGTGCCCCGGAGCTTCT	N/A N/A	60	65	[43]	NM_205209.1
GLUT2	Glucose transporter-2 (SLC2A2)	F-TGATCGTGGCACTGATGGTT R-CCACCAGGAAGAC^↓^GGAGATA	N/A 8/9	60	171	This study	NM_207178.1
LAT1	L type amino acid transporter-1 (SLC7A5)	F-GATTGCAACGGGTGATGTGA R- CCCCACACCCACTTTTGTTT	N/A N/A	60	70	[40]	KT876067.1
PGA5	Pepsinogen A	F-TCCGTCTACCTGAGCAA^↓^GGAT R- AAGCAGGCGACGTACTTGTT	6–7 N/A	60	167	This study	NM_204878.1
PGC	Pepsinogen C	F-ATCGGGATTGAGGA^↓^CTTCGC R- TGAAGACCTGGTTGGGAACG	6–7 N/A	60	115	This study	NM_204877.2
PepT1	Peptide transporter-1 (SLC15A1)	F-TACGCATACTGTCACCATCA R-TCCTGAGAACGGACTGTAAT	N/A N/A	60	205	[44]	AY029615.1
PepT2	Peptide transporter-2 (SLC15A2)	F-TGACTGGGCATCGGAACAA R-ACCCGTGTCACCATTTTAACCT	N/A N/A	60	63	[41]	NM_001319028.1
PNLIP	Pancreatic lipase	F-GCATCTGGGAAG^↓^GAACTAGGG R- TGAACCACAAGCATAGCCCA	7/8 N/A	60	113	This study	NM_001277382.1
rBAT	Solute carrier family 3, member1 (SLC3A1)	F-CCCGCCGTTCAACAAGAG R- AATTAAATCCATCGACTCCTTTGC	N/A N/A	60	70	[40]	XM_426125.4
SI	Sucrase isomaltase	F-GCTTTAAG^↓^ATGGGCAAGAGGAAG R- CCACCACCAGGCAAAAGAGG	1/2 N/A	60	65	This study	XM_015291762.1
y^+^LAT1	y^+^ L amino acid transporter-1 (SLC7A7)	F-TACTGAGGCTGACTGGAGGAA R- ACGACGTACAGCACAAT^↓^ATCTGG	N/A 1/2	62	227	This study	XM_418326.5
y^+^LAT2	y^+^ L amino acid transporter-2 (SLC7A6)	F-GCCCTGTCAGTAAATCAGACAAGA R-TTCAGTTGCATTGTGTTTTGGTT	N/A N/A	60	82	[40]	NM_001005832.1
HPRT1	Hypoxanthine Phosphoribosyltransferase 1	F-ACTGGCTGCTTCTTGTG R-GGTTGGGTTGTGCTGTT	N/A N/A	63	245	[45]	NM_204848.1
TBP	TATA-Box binding protein	F-TAGCCCGATGATGCCGTAT R- GTTCCCTGTGTCGCTTGC	N/A N/A	62	147	[46]	NM_205103 D83127

N/A means not applicable

### Real-time quantitative PCR (qPCR)

Quantitative PCR was performed in triplicates using a SYBR Green kit SensiFAST™ SYBR® No-ROX (Bioline, Sydney, Australia) with Rotorgene 6000 real-time PCR machine (Corbett Research, Sydney, Australia). The PCR reaction was performed in a volume of 10 μL containing 5 μL of 2× SensiFAST, 400 mM of each primer and 2 μL of cDNA template. After thermal cycling, amplification cycle (Cq) values for all genes were collected and imported into qBase+ version 3.0 (Biogazelle, Zwijnbeke, Belgium) software and analyzed against two optimized reference genes, HPRT1 and TBP, in the current study. Both reference genes were applied to the samples of each tissue. The qBase+ applied an arithmetic mean method to transform logarithmic Cq value to linear relative quantity using exponential function for relative quantification of genes [22, 23] and the output data was exported to SPSS statistics version 22 (IBM SPSS, UK) for further analysis. The relative expression levels of the genes in respective treatment groups are expressed as means of normalized relative quantities (NRQ). Relative quantity for individual genes was scaled to the lowest average expression level of the treatment being 1.

The genes analyzed in the tissues are listed as follow: PGA5, PGC and CCK, in proventriculus; AMY2A, ATP1A1, CCK1R, CCK, CELA1, CELA2A, PNLIP, in pancreas; and APN, ASCT1, ATP1A1, B^o^AT, CAT1, CAT2, CCK1R, CCK, EAAT3, b^o,+^AT, GLUT1, GLUT2, LAT1, PepT1, PepT2, SI, y^+^LAT1, y^+^LAT2, and rBAT in the intestine.

### Statistical analyses

The data were analyzed using the General Linear Models (GLM) procedure of SPSS statistics version 22 (IBM SPSS, UK) for the main effect of particle sizes, and SB supplementation along with their interactions. The main effect was analyzed with a treatment with pooled values against the other treatment. Differences between mean values were determined using LSD test at the level of *P* < 0.05. Correlations between FCR of the grower phase, d 10–24, and the expression levels of the genes were conducted using the procedures of SPSS statistics version 22 (IBM SPSS, UK). For correlation analysis, FCR was calculated in each pen (*n* = 14 birds) and the mRNA expression of the proventricular pepsinogens was measured from a randomly selected bird in each pen (n = 1 bird).

## Results

### Performance and gizzard weight

The effect of corn particle size and SB on broiler performance is presented in Table 3. On d 10, weight gain and feed intake were significantly higher in birds fed finely ground corn (FC) than those fed coarsely ground corn (CC; *P* < 0.05). FCR was impaired in birds fed the diet containing 2% SB compared to those fed the diet without SB treatment during d 0–10 (*P* < 0.05). No interaction was observed between particle size and SB in early age (*P* > 0.05). During d 10–24, significant particle size × SB interactions were observed for FCR (*P* < 0.01). The birds fed CC with 2% SB had lower FCR than those fed CC without SB. However, this was not the case when fine corn was fed. Similarly, CC reduced FCR compared to FC only when 2% SB was included in the diet. During d 10–24, birds fed SB were heavier than those fed without SB (*P* < 0.05).

**Table 3:** Effect of particle size and sugar cane bagasse on broilers growth performance^1^

Treatments			D0–10			D10–24	
Particle size	SB	FCR	Weight gain (g/bird)	Feed intake (g/bird)	FCR	Weight gain (g/bird)	Feed intake (g/bird)
CC	0%	1.049	277	290	1.332^a^	1158	1542
FC	0%	1.053	279	294	1.333^a^	1181	1573
CC	2%	1.072	267	287	1.280^b^	1233	1579
FC	2%	1.074	281	302	1.351^a^	1189	1606
Main effect							
SB							
0%		1.050^b^	278	292	1.332	1169^b^	1557
2%		1.073^a^	274	294	1.316	1211^a^	1593
Particle size							
CC		1.061	272^b^	288^b^	1.306^b^	1196	1560
FC		1.063	280^a^	298^a^	1.342^a^	1185	1590
*P* value							
SB		0.005	0.279	0.557	0.143	0.037	0.171
Particle size		0.713	0.020	0.019	0.003	0.582	0.251
SB × particle size		0.877	0.097	0.134	0.004	0.090	0.940

^1^Within a column, values with different superscripts are significantly different from each other at P < 0.05

The effect of corn particle size and SB on relative gizzard weight is presented in Table 4. On d 24, the broilers fed CC had heavier gizzards (P < 0.05) compared to those fed FC. A particle size × SB interaction was observed for relative gizzard weight (P < 0.05). Addition of 2% SB increased the relative gizzard weight only in the FC fed birds.

**Table 4:** Effect of particle size and sugar cane bagasse on relative gizzard weight at d 24^1^

Treatments		Relative gizzard weight
Particle size	SB	
CC	0%	1.934^a^
FC	0%	1.713^b^
CC	2%	1.921^a^
FC	2%	1.922^a^
Main effect		
SB		
0%		1.823
2%		1.922
Particle size		
CC		1.927^a^
FC		1.817^b^
P value		
SB		0.060
Particle size		0.037
SB × particle size		0.036

^1^Within a column, values with different superscripts are significantly different from each other at P < 0.05

### Upregulation of pepsinogen a and C in proventriculus by sugarcane bagasse and coarsely ground corn

The mRNA expression of three genes was investigated in response to SB addition and corn particle size in the proventriculus as presented in Table 5. The mRNA expression of CCK was not affected by SB addition or corn particle size. A particle size × SB interaction was observed in the expression of genes PGA5 (A) and PGC (C; *P* < 0.01). The combination of SB and CC significantly upregulated PGA5 and PGC compared to the expression of genes in the other three groups. Both pepsinogen A (P < 0.01 and *R* = − 0.53) and C (P < 0.01 and *R* = − 0.59) were negatively correlated to FCR on d 24.

**Table 5:** Effect of particle size and sugar cane bagasse on expression of proventricular genes at d 24^1,2^

Treatments		PGA5	PGC	CCK
Particle size	SB			
CC	0%	1.096^b^	1.111^b^	1.000
FC	0%	1.361^b^	1.241^b^	1.391
CC	2%	2.712^a^	2.984^a^	1.066
FC	2%	1.000^b^	1.000^b^	1.217
Main effect				
SB				
0%		1.228	1.175	1.196
2%		1.855	1.992	1.142
Particle size				
CC		1.903^a^	2.048^a^	1.034
FC		1.180^b^	1.120^b^	1.304
P value				
SB		0.070	0.078	0.811
Particle size		0.039	0.047	0.239
SB × particle size		0.007	0.026	0.598

^1^The relative expression levels of the genes of respective treatment groups are expressed as means of normalized relative quantities (NRQ). Relative quantities for individual genes are scaled to the average across all unknown samples per target gene

^2^Within a column, values with different superscripts are significantly different from each other at *P* < 0.05

### Upregulation of pancreatic AMY2A and CELA1 by sugarcane bagasse

The mRNA expression of seven pancreatic genes in response to feed SB addition and corn particle size was examined (Table 6). Genes AMY2A and CELA1 were upregulated by 2% SB addition to the diets (*P* < 0.05), while no response to SB was observed in other genes investigated, namely, ATP1A1, CCK1R, CCK, CELA2A, and PNLIP. Corn particle size did not affect the expression of pancreatic genes either as a main effect and no interactions were observed (*P* > 0.05).

**Table 6:** Effect of particle size and sugar cane bagasse on expression of pancreatic genes at d 24^1,2^

Treatments		AMY2A	ATP1A1	CCK1R	CCK	CELA1	CELA2A	PNLIP
Particle size	SB							
CC	0%	1.042	1.230	1.231	1.000	1.173	1.460	1.432
FC	0%	1.314	1.264	1.254	1.091	1.089	1.401	1.226
CC	2%	1.559	1.313	1.384	1.492	1.599	1.171	1.177
FC	2%	1.500	1.000	1.375	1.143	1.678	1.185	1.257
Main effect								
SB								
0%		1.178^b^	1.248	1.243	1.046	1.131^b^	1.431	1.329
2%		1.530^a^	1.157	1.380	1.318	1.639^a^	1.178	1.217
Particle size								
CC		1.301	1.272	1.307	1.246	1.386	1.316	1.304
FC		1.407	1.133	1.315	1.118	1.384	1.293	1.242
*P* value								
SB		0.024	0.543	0.398	0.162	0.040	0.115	0.428
Particle size		0.468	0.355	0.964	0.498	0.991	0.885	0.656
SB × particle size		0.263	0.252	0.922	0.253	0.724	0.813	0.317

^1^The relative expression levels of the genes of respective treatment groups are expressed as means of normalized relative quantities (NRQ). Relative quantities for individual genes are scaled to the average across all unknown samples per target gene

^2^Within a column, values with different superscripts are significantly different from each other at P < 0.05

### Upregulation of APN by coarsely ground corn and CAT1 by sugarcane bagasse in duodenum

The mRNA expression of nineteen genes was investigated in response to feed SB addition and corn particle size in the duodenum as presented in Tables 7 and 8. The CC diet significantly upregulated APN in the duodenum compared with expression of the gene in the FC diet group. No responses to CC diet were observed for other genes investigated, namely, ASCT1, ATP1A1, B^o^AT, CAT1, CAT2, CCK1R, CCK, b^o,+^AT, EAAT3, GLUT1, GLUT2, LAT1, PepT1, PepT2, SI, y^+^LAT2, and rBAT. Addition of 2% SB upregulated CAT1 in the duodenum compared to the expression in the birds without SB (*P* < 0.05). However, 2% SB did not affect the expression of the genes APN, ASCT1, ATP1A1, B^o^AT, CAT2, CCK1R, CCK, b^o,+^AT, EAAT3, GLUT1, GLUT2, LAT1, PepT1, PepT2, SI, y^+^LAT2, and rBAT in the duodenum (*P* > 0.05). A particle size × SB interaction was observed for expression of gene y^+^LAT1 (*P* < 0.05), where y^+^LAT1 was downregulated by CC only in the birds fed diet without SB supplementation, but no significant difference was observed for the expression of the gene between FC and CC groups when 2% SB was added.

**Table 7:** Effect of particle size and sugar cane bagasse on expression of duodenal genes at d 24^1,2^

Treatments		APN	ASCT1	ATP1A1	B^o^AT	CAT1	CAT2	CCK1R	CCK	EAAT3
Particle size	SB									
CC	0%	1.867	1.330	1.000	1.199	1.132	1.198	1.588	1.049	1.000
FC	0%	1.149	1.000	1.009	1.238	1.000	1.338	1.590	1.304	1.212
CC	2%	1.750	1.245	1.228	1.000	1.615	1.000	1.000	1.024	1.174
FC	2%	1.000	1.276	1.059	1.212	1.707	1.100	1.543	1.051	1.082
Main effect										
SB										
0%		1.507	1.165	1.004	1.219	1.066^b^	1.267	1.588	1.177	1.106
2%		1.375	1.261	1.144	1.106	1.661^a^	1.049	1.271	1.037	1.128
Particle size										
CC		1.809^a^	1.288	1.114	1.100	1.374	1.099	1.294	1.036	1.087
FC		1.074^b^	1.138	1.034	1.224	1.353	1.218	1.566	1.178	1.147
P value										
SB		0.689	0.419	0.203	0.444	0.037	0.088	0.202	0.508	0.924
Particle size		0.036	0.210	0.465	0.395	0.940	0.338	0.273	0.501	0.787
SB × particle size		0.961	0.135	0.411	0.556	0.675	0.870	0.273	0.587	0.499

^1^The relative expression levels of the genes of respective treatment groups are expressed as means of normalized relative quantities (NRQ). Relative quantities for individual genes are scaled to the average across all unknown samples per target gene

^2^Within a column, values with different superscripts are significantly different from each other at P < 0.05

**Table 8:** Effect of particle size and sugar cane bagasse on expression of duodenal genes at d 24^1,2^

Treatments		b^o,+^AT	rBAT	GLUT1	GLUT2	LAT1	PepT1	PepT2	SI	y^+^LAT1	y^+^LAT2
Particle size	SB										
CC	0%	1.000	1.003	1.283	1.517	1.640	1.000	1.981	1.000	1.000^b^	1.063
FC	0%	1.204	1.175	1.144	1.110	1.000	1.126	1.380	1.189	1.484^b^	1.046
CC	2%	1.057	1.086	1.263	1.223	1.455	1.289	1.351	1.243	1.241^ab^	1.179
FC	2%	1.195	1.000	1.000	1.000	1.456	1.041	1.233	1.118	1.044^ab^	1.000
Main effect											
SB											
0%		1.101	1.089	1.213	1.313	1.320	1.063	1.681	1.095	1.241	1.055
2%		1.127	1.043	1.131	1.112	1.456	1.165	1.292	1.181	1.143	1.090
Particle size											
CC		1.029	1.045	1.273	1.371	1.548	1.144	1.666	1.121	1.121	1.121
FC		1.200	1.088	1.071	1.055	1.229	1.083	1.307	1.153	1.264	1.024
P value											
SB		0.875	0.746	0.541	0.389	0.424	0.579	0.323	0.618	0.545	0.822
Particle size		0.289	0.762	0.142	0.183	0.069	0.741	0.360	0.853	0.382	0.530
SB × particle size		0.837	0.367	0.643	0.691	0.068	0.314	0.536	0.370	0.047	0.602

^1^The relative expression levels of the genes of respective treatment groups are expressed as means of normalized relative quantities (NRQ). Relative quantities for individual genes are scaled to the average across all unknown samples per target gene

^2^Within a column, values with different superscripts are significantly different from each other at P < 0.05

### Upregulation of jejunal ASCT1 and y^+^LAT2 by coarsely ground corn and CAT1 by sugarcane bagasse and downregulation of jejunal GLUT2 by sugarcane bagasse

The mRNA expression of nineteen jejunal genes in response to feed SB addition and corn particle size was examined (Tables 9 and 10). The expression of ASCT1 (*P* < 0.01) and y^+^LAT2 (*P* < 0.05) in jejunum were upregulated in birds fed CC diet compared with those fed FC diet. Corn particle size did not affect other genes investigated: APN, ATP1A1, B^o^AT, CAT1, CAT2, CCK1R, CCK, b^o,+^AT, EAAT3, GLUT1, GLUT2, LAT1, PepT1, PepT2, SI, y^+^LAT1, and rBAT, in jejunum (*P* > 0.05). Birds fed 2% SB had upregulated jejunal CAT1 and downregulated GLUT2 compared to birds fed diet without SB (P < 0.05). No responses to 2% SB were observed in other investigated genes: APN, ATP1A1, B^o^AT, CAT2, CCK1R, CCK, b^o,+^AT, EAAT3, GLUT1, LAT1, PepT1, PepT2, SI, y^+^LAT1, and rBAT, in jejunum (P > 0.05). No interactions were observed between particle size and SB on expression of other investigated genes in the jejunum. However, a tendency for a particle size × SB interaction was observed for expression of CAT1 (*P* = 0.077) in the jejunum, where the birds fed CC diet with 2% SB inclusion tended to upregulate CAT1.

**Table 9:** Effect of particle size and sugar cane bagasse on expression of jejunal genes at d 24^1,2^

Treatments		APN	ASCT1	ATP1A1	B^o^AT	CAT1	CAT2	CCK1R	CCK	EAAT3
Particle size	SB									
CC	0%	1.071	1.386	1.206	1.058	1.000	1.025	1.004	1.136	1.188
FC	0%	1.103	1.000	1.083	1.062	1.099	1.113	1.066	1.163	1.124
CC	2%	1.148	1.222	1.196	1.037	3.236	1.000	1.048	1.000	1.083
FC	2%	1.000	1.197	1.000	1.137	1.451	1.126	1.021	1.134	1.000
Main effect										
SB										
0%		1.086	1.192	1.145	1.060	1.050^b^	1.069	1.035	1.149	1.156
2%		1.074	1.155	1.098	1.087	2.343^a^	1.063	1.034	1.066	1.041
Particle size										
CC		1.109	1.304^a^	1.202	1.048	2.119	1.013	1.026	1.067	1.136
FC		1.051	1.044^b^	1.041	1.099	1.276	1.119	1.044	1.148	1.062
P value										
SB		0.917	0.628	0.705	0.873	0.019	0.943	0.993	0.586	0.428
Particle size		0.644	0.003	0.199	0.762	0.111	0.232	0.886	0.596	0.611
SB × particle size		0.472	0.117	0.765	0.778	0.077	0.827	0.718	0.726	0.948

^1^The relative expression levels of the genes of respective treatment groups are expressed as means of normalized relative quantities (NRQ). Relative quantities for individual genes are scaled to the average across all unknown samples per target gene

^2^Within a column, values with different superscripts are significantly different from each other at P < 0.05

**Table 10:** Effect of particle size and sugar cane bagasse on expression of jejunal genes at d 24^1,2^

Treatments		b^o,+^AT	rBAT	GLUT1	GLUT2	LAT1	PepT1	PepT2	SI	y^+^LAT1	y^+^LAT2
Particle size	SB										
CC	0%	1.091	1.157	1.212	2.630	1.327	1.188	2.087	1.479	1.017	1.276
FC	0%	1.202	1.108	1.130	1.871	1.000	1.164	1.659	1.199	1.048	1.098
CC	2%	1.049	1.000	1.278	1.427	1.190	1.078	1.633	1.149	1.011	1.148
FC	2%	1.303	1.006	1.000	1.000	1.122	1.000	1.611	1.000	1.012	1.000
Main effect											
SB											
0%		1.147	1.132	1.170	2.243^a^	1.164	1.175	1.873	1.339	1.032	1.186
2%		1.176	1.003	1.139	1.214^b^	1.155	1.039	1.622	1.075	1.011	1.075
Particle size											
CC		1.070	1.078	1.245	2.028	1.258	1.132	1.849	1.315	1.014	1.212^a^
FC		1.253	1.057	1.064	1.435	1.060	1.082	1.646	1.100	1.030	1.049^b^
P value											
SB		0.902	0.395	0.744	0.018	0.968	0.389	0.649	0.089	0.824	0.119
Particle size		0.452	0.886	0.075	0.157	0.309	0.747	0.713	0.163	0.865	0.028
SB × particle size		0.769	0.857	0.322	0.684	0.501	0.864	0.682	0.660	0.868	0.837

^1^The relative expression levels of the genes of respective treatment groups are expressed as means of normalized relative quantities (NRQ). Relative quantities for individual genes are scaled to the average across all unknown samples per target gene

^2^Within a column, values with different superscripts are significantly different from each other at P < 0.05

### Upregulation of ileal PepT2 by coarsely ground corn and CAT1, B^o^AT and ATP1A1 by sugarcane bagasse

The mRNA expression of eighteen ileal genes in response to feed SB addition and corn particle size was examined (Tables 11 and 12). Birds fed the CC diet had upregulated ileal PepT2 compared to birds fed the FC diet (*P* < 0.05). The CC diet tended to upregulate CCK1R (*P* = 0.056) and y^+^LAT1 (*P* = 0.097) in the ileum. Corn particle size did not affect the expression of: APN, ASCT1, ATP1A1, B^o^AT, CAT1, CAT2, b^o,+^AT, EAAT3, GLUT1, GLUT2, LAT1, PepT1, SI, y^+^LAT2 and rBAT, in the ileum. Inclusion of 2% SB upregulated expression of B^o^AT and CAT1 in the ileum compared to birds given no SB (P < 0.05). Inclusion of 2% SB had no effect (*P* > 0.05) on other genes: APN, ASCT1, CAT2, CCK1R, b^o,+^AT, EAAT3, GLUT1, GLUT2, LAT1, PepT1, PepT2, SI, y^+^LAT1, y^+^LAT2, and rBAT in ileum. A particle size × SB interaction was observed for expression of ATP1A1 in the ileum (P < 0.05), where 2% SB inclusion increased mRNA expression of ATP1A1 in the birds fed the CC diet but not in the birds fed the FC diet.

**Table 11:** Effect of particle size and sugar cane bagasse on expression of ileal genes at d 24^1,2^

Treatments		APN	ASCT1	ATP1A1	B^o^AT	CAT1	CAT2	CCK1R	EAAT3
Particle size	SB								
CC	0%	1.219	1.153	1.000^b^	1.000	1.053	1.077	1.240	1.204
FC	0%	1.550	1.060	1.174^ab^	1.003	1.000	1.026	1.000	1.069
CC	2%	1.000	1.000	1.193^a^	1.165	1.626	1.000	1.358	1.000
FC	2%	1.364	1.171	1.096^ab^	1.251	2.216	1.016	1.009	1.372
Main effect									
SB									
0%		1.385	1.107	1.087	1.002^b^	1.027^b^	1.052	1.120	1.137
2%		1.182	1.085	1.145	1.208^a^	1.921^a^	1.008	1.184	1.187
Particle size									
CC		1.110	1.077	1.097	1.082	1.340	1.038	1.299	1.102
FC		1.457	1.115	1.136	1.128	1.608	1.021	1.004	1.221
P value									
SB		0.401	0.867	0.387	0.019	0.029	0.627	0.664	0.876
Particle size		0.156	0.765	0.554	0.583	0.489	0.846	0.056	0.709
SB × particle size		0.943	0.314	0.049	0.611	0.407	0.705	0.707	0.428

^1^The relative expression levels of the genes of respective treatment groups are expressed as means of normalized relative quantities (NRQ). Relative quantities for individual genes are scaled to the average across all unknown samples per target gene

^2^Within a column, values with different superscripts are significantly different from each other at P < 0.05

**Table 12:** Effect of particle size and sugar cane bagasse on expression of ileal genes at d 24^1,2^

Treatments		b^o,+^AT	rBAT	GLUT1	GLUT2	LAT1	PepT1	PepT2	SI	y^+^LAT1	y^+^LAT2
Particle size	SB										
CC	0%	1.000	1.000	1.269	1.026	1.205	1.028	3.520	1.241	1.322	1.124
FC	0%	1.040	1.136	1.140	1.000	1.164	1.000	1.426	1.392	1.000	1.073
CC	2%	1.110	1.013	1.141	1.244	1.000	1.221	3.224	1.000	1.269	1.000
FC	2%	1.123	1.015	1.000	1.260	1.277	1.197	1.984	1.265	1.102	1.198
Main effect											
SB											
0%		1.021	1.068	1.205	1.013	1.185	1.014	2.473	1.316	1.160	1.099
2%		1.116	1.014	1.071	1.252	1.139	1.209	2.604	1.133	1.185	1.100
Particle size											
CC		1.056	1.007	1.206	1.135	1.103	1.125	3.372^a^	1.121	1.295	1.063
FC		1.081	1.076	1.071	1.130	1.220	1.099	1.705^b^	1.329	1.051	1.136
P value											
SB		0.474	0.554	0.479	0.369	0.787	0.141	0.857	0.534	0.862	0.994
Particle size		0.844	0.452	0.477	0.986	0.491	0.842	0.031	0.481	0.097	0.525
SB × particle size		0.918	0.460	0.975	0.936	0.355	0.988	0.557	0.846	0.589	0.289

^1^The relative expression levels of the genes of respective treatment groups are expressed as means of normalized relative quantities (NRQ). Relative quantities for individual genes are scaled to the average across all unknown samples per target gene

^2^Within a column, values with different superscripts are significantly different from each other at P < 0.05

## Discussion

This study investigated responses of mRNA expression of genes encoding digestive enzymes and nutrient transporters in birds fed diets with SB and/or increased corn particle size. Gene expression in the proventriculus, pancreas and intestine of broiler chickens was increased in line with bird performance and gizzard development. Addition of 2% SB and inclusion of CC particle in feed upregulated some genes encoding digestive enzymes, and nutrient transporters together with improved gizzard function and performance in birds up to d 24.

The combination of CC and SB improved broiler performance as demonstrated by lower FCR in birds fed CC together with SB. Coarse particle size and structural fiber may have: 1) extended digesta retention time, leading to prolonged exposure of nutrients to endogenous enzymes; 2) promoted gut reflux, re-exposing undigested nutrients to digestive enzymes for better digestion; 3) created a better microenvironment for enzyme activity around coarse corn particles of SB that has a strong water-holding capacity [24]; 4) enhanced gizzard activity, leading to secretion of more digestive juices and better ability to grind feed particles. Indeed, both pepsinogen A and C were correlated with FCR on d 24. The significantly greater mRNA expression of pepsinogen A and C in the birds fed CC diet supplemented with SB might increase the production of pepsin in proventriculus and improved FCR. The similarity in increase between mRNA of pepsinogen A and that of pepsinogen C suggests that the mechanism of stimulation of the two zymogens was similar and could have been caused by; physical effects of the combination of CC and SB on the oxyntic (or oxynticopeptic) cells of the proventriculus or, by one or both of the following possible mechanisms that may affect pepsinogen production: 1) the stimulating effect of coarse particles and fiber on gizzard function, in particular more frequent and powerful contractions which may potentially reflux the digesta back into the proventriculus repeatedly during each gizzard contraction and thereby allows for more proventricular secretions and thus re-expose the digesta to these two zymogens and pepsin; 2) Coarse particle and SB might cause more rapid duodenal-gizzard reflux and move the digesta back to the gizzard and then to proventriculus. Indeed, bile salts, associated with duodenum, have been reported to be found in the gizzard at low concentrations [25]. It has been hypothesized that the structural components, such as whole or coarsely ground cereals, or fiber materials, in the diet induce an increase in gastrointestinal reflux via a well-developed gizzard [25, 26]. However, in this study, the SB may have created an environment where gizzard contractions were enhanced and mucosal surface cleaned, leading to unhindered duodenal-gizzard reflux. This, in turn, could expose digesta to proventricular zymogens and enzymes repeatedly, resulting in better digestion of nutrients and a more dynamic foregut.

In recent years, the beneficial effects of fiber on gastrointestinal tract development and nutrient utilization in poultry have been discussed frequently. The source, physical structure and amount of fiber in the diet determine the effectiveness of fiber on birds [27, 28]. For instance, previous studies have reported that insoluble fiber (ARBOCEL^®^) increased the activity of pancreatic enzymes such as chymotrypsin [29, 30]. Furthermore, it has been suggested that oat hulls may stimulate pancreatic secretion of amylase and thus increase the activity of amylase in the jejunum [17]. The significantly greater expression of pancreatic AMY2A and CELA1 in the birds fed diet supplemented with SB is at least partially responsible for better performance in the present study. A rapid and noticeable enlargement in gizzard size was observed when structural components were included in the diet. The increase in the gizzard weight is a logical consequence of an increased need for particle size reduction, as the increased mechanical grinding activity of gizzard increases the size of the two pairs of gizzard muscles [9]. In the present study, gizzard weight was the lowest in the FC diet group, this may be due to the fact that the birds are not required to cope with the extra grinding demand from feed with fine particles prior to passing through the pyloric sphincter to the intestine. A number of studies have suggested that well developed gizzards in birds fed diets with large ingredient particles or fiber, improve gut motility, and thus digestibility and performance due to increased releases of cholecystokinin (CCK) which stimulates pancreatic enzyme secretion and gastro-duodenal reflux [3, 9, 17, 29, 31]. Although the expression of pancreatic AMY2A and CELA1 was upregulated by 2% SB, the expression of both CCK and its receptor (CCK1R) in different tissues was not affected by particle size and SB addition in this study. It has been previously reported that CCK at physiological concentrations has no influence on pancreatic secretion from isolated pancreatic acini in the chicken [32]. It has also been stated that CCK plays a more important role as a pancreas-stimulating hormone in mammals than in birds [32]. Furthermore, it was reported that the regulation of pancreatic secretion is controlled by numerous hormones such as melatonin and glucagon, regulatory peptides including C-natriuretic peptide, and neurotransmitters such as serotonin, vasoactive intestinal peptide (VIP) and gastrin releasing peptide (GIP) [33]. Therefore, the mechanism underlying the heightened secretion of pancreatic enzymes by inclusion of fiber or large feed particles in chicken diets is not well understood and further investigation is warranted.

Several studies investigating structural components of diets, such as coarse particle size or fiber, have shown improved nutrient digestibility [3, 14–17, 34] via increased digesta retention time. Reports investigating the impact of corn particle size and fiber on digestive enzymes and nutrient transporters are scant or non-existent. In the present study, broiler performance was improved by inclusion of dietary coarse particle grain and SB and various nutrient transporters and digestive enzymes in the duodenum, jejunum and ileum were shown to be upregulated. For instance, B^0^AT, the neutral amino acid transporter located at the brush border membrane, in ileum and CAT1, a transporter mediating the bidirectional transport of cationic amino acids, in duodenum, jejunum and ileum were upregulated by SB. Furthermore, duodenal APN, responsible for final digestion of peptides by N terminus cleavage, jejunal ASCT1, responsible for Na + −dependent neutral amino acid transporter, and jejunal y^+^LAT2, responsible for Na + −dependent neutral/cationic amino acid exchanger, and ileal PepT2, which has a minor contribution in transporting di- and tri-peptides, were upregulated by CC. In fact, the upregulated nutrient transporters will not only improve nutrient absorption but also play a vital role in the maintenance of intestinal barrier integrity and immune response. A deficiency of amino acids, such as alanine, cysteine, serine, threonine, arginine, and lysine, has long been known to impair immune function and increase the susceptibility of animals to infectious disease [35]. Different mechanisms can be involved to elucidate the increase in expression of amino acid transporters in the gut. For example, CAT-1 mRNA expression level varies considerably in different tissues and cell types and can be modulated by a variety of stimuli, including cell proliferation, growth factors, cytokines, hormones, and nutrients [36]. In the current study, a well-developed gizzard may generate stronger reverse peristalsis contractions that may stimulate the secretion of digestive enzymes and enzyme precursors in both the proventriculus and pancreas and consequently produce higher levels of substrates of nutrient transporters and thus upregulate those nutrient transporters in the gut. In general, three distinct sites of reverse peristalsis can be observed in the gastrointestinal tract of birds [37]: 1) gastric reflux which transfer the digesta from gizzard to proventriculus via gastroduodenal contractions and this contraction cycle takes place 2–4 times per min.; 2) the small intestinal reflux which transfers digesta from the duodenum and jejunum into the gastric area and occurs about 4 times per 60 min.; 3) cloaca-cecal reflux, which transfers urinary nitrogen to the ceca via the colon [38]. It has been well documented that structural components of the diet such as fiber and coarse or whole cereals enhance the gut motility and thereby increase the digesta retention time and better bird performance [26, 39]. Therefore, SB and CC may have increased gut motility and digesta retention time, particularly in the upper part of digestive tract, and thereby enhanced the production of digestive enzymes, enzyme precursors and nutrient transporters. These active functional proteins promote digestion of nutrients and thus the growth and feed conversion efficiency of the birds.

## Conclusions

In conclusion, the inclusion of either SB or CC in a pelleted diet is beneficial to the birds by improving performance likely through the upregulation of genes encoding digestive enzymes and nutrient transporters. The combination of CC and SB was more beneficial for the upregulation of some genes such as PGA5 and PGC. The results suggest enhanced gizzard development as a mode of action for higher production of digestive enzymes and nutrient transporters. These findings provide insights on how dietary fiber and particle size independently or in combination can improve bird performance based on the analysis of gene expression. The knowledge obtained herein will be useful to understand the underlying mechanisms of how feed additives can improve nutrient digestibility and thus feed efficiency. Further, the outcomes lay a foundation for future research to elucidate the usefulness of fiber supplementation and coarse particle inclusion in feed in a nutrigenomic way.

## Abbreviations

AMY2A: pancreatic alpha 2A amylase
APN: aminopeptidase N
ASCT1: alanine, serine, cysteine, and threonine transporter
ATP1A1: ATPase Na+/K+ transporting subunit alpha 1
B^0^AT: solute carrier family 6, member 19
b^o,+^AT: solute carrier family 7, member 9
CAT1: cationic amino acid transporter-1
CAT2: cationic amino acid transporter-2
CC: coarsely ground corn
CCK: cholecystokinin
CCK1R: cholecystokinin type 1 receptor
CELA1: chymotrypsin-like elastase family, member 1
CELA2A: chymotrypsin like elastase family member 2A
EAAT3: excitatory amino acid transporter 3
FC: finely ground corn
FCR: feed conversion ratio
GIP: gastrin releasing peptide
GIT: gastrointestinal tract
GLUT1: glucose transporter-1
GLUT2: glucose transporter-2
GMD: geometric mean diameter
HPRT1: hypoxanthine Phosphoribosyltransferase 1
LAT1: L type amino acid transporter-1
NSP: non-starch polysaccharides
PepT1: peptide transporter-1
PepT2: peptide transporter-2
PGA5: pepsinogen A
PGC: pepsinogen C
PNLIP: pancreatic lipase
rBAT: solute carrier family 3, member1
SB: sugarcane bagasse
SI: sucrase isomaltase
SID: standard ileal digestible
TBP: TATA-Box binding protein
VIP: vasoactive intestinal peptide
y^+^LAT1: y^+^ L amino acid transporter-1
y^+^LAT2: y^+^ L amino acid transporter-2

## References

[1] Hofacre C, Beacorn T, Collett S, Mathis G (2003). Using competitive exclusion, mannan-oligosaccharide and other intestinal products to control necrotic enteritis. J Appl Poult Res.

[2] Caly DL, D'Inca R, Auclair E, Drider D (2015). Alternatives to antibiotics to prevent necrotic enteritis in broiler chickens: a microbiologist's perspective. Front Microbiol.

[3] Kheravii SK, Swick RA, Choct M, Wu S-B. Coarse particle inclusion and lignocellulose-rich fiber addition in feed benefit performance and health of broiler chickens. Poult Sci. 2017a; 10.3382/ps/pex123. 10.3382/ps/pex123 28637332

[4] Zaefarian F, Abdollahi M, Ravindran V (2016). Particle size and feed form in broiler diets: impact on gastrointestinal tract development and gut health. Worlds Poult Sci J..

[5] Hetland H, Svihus B, Choct M (2005). Role of insoluble fiber on gizzard activity in layers. J Appl Poult Res.

[6] Duke G. Alimentary canal: secretion and digestion, special digestive functions, and absorption. Edtion ed. Avian physiology. New York, NY: Springer, 1986:289–302.

[7] Guinotte F, Gautron J, Nys Y, Soumarmon A (1995). Calcium solubilization and retention in the gastrointestinal tract in chicks (Gallus domesticus) as a function of gastric acid secretion inhibition and of calcium carbonate particle size. Br J Nutr.

[8] Sklan D, Shachaf B, Baron J, Hurwitz S (1978). Retrograde movement of digesta in the duodenum of the chick: extent, frequency, and nutritional implications. J Nutr.

[9] Svihus B (2011). The gizzard: function, influence of diet structure and effects on nutrient availability. Worlds Poult Sci J.

[10] Sacranie A, Svihus B, Denstadli V, Moen B, Iji P, Choct M (2012). The effect of insoluble fiber and intermittent feeding on gizzard development, gut motility, and performance of broiler chickens. Poult Sci.

[11] Washburn K (1991). Efficiency of feed utilization and rate of feed passage through the digestive system. Poult Sci.

[12] Röder PV, Geillinger KE, Zietek TS, Thorens B, Koepsell H, Daniel H (2014). The role of SGLT1 and GLUT2 in intestinal glucose transport and sensing. PLoS One.

[13] Fotiadis D, Kanai Y, Palacín M (2013). The SLC3 and SLC7 families of amino acid transporters. Mol Asp Med.

[14] Jiménez-Moreno E, de Coca-Sinova A, González-Alvarado J, Mateos G. Inclusion of insoluble fiber sources in mash or pellet diets for young broilers. 1. Effects on growth performance and water intake. Poult Sci. 2016:41–52. 10.3382/ps/pev309 26574033

[15] Xu Y, Stark C, Ferket P, Williams C, Auttawong S, Brake J (2015). Effects of dietary coarsely ground corn and litter type on broiler live performance, litter characteristics, gastrointestinal tract development, apparent ileal digestibility of energy and nitrogen, and intestinal morphology. Poult Sci.

[16] Xu Y, Stark C, Ferket P, Williams C, Pacheco W, Brake J (2015). Effect of dietary coarsely ground corn on broiler live performance, gastrointestinal tract development, apparent ileal digestibility of energy and nitrogen, and digesta particle size distribution and retention time. Poult Sci.

[17] Hetland H, Svihus B, Krogdahl A (2003). Effects of oat hulls and wood shavings on digestion in broilers and layers fed diets based on whole or ground wheat. Br Poult Sci.

[18] Aviangen. Ross 308 broiler performance objectives. 2014; http:// http://en.aviagen.com/assets/Tech_Center/Ross_Broiler/Ross-Broiler-Handbook-2014i-EN.pdf. Accessed 13 Mar 2018.

[19] Englyst HN, Quigley ME, Hudson GJ (1994). Determination of dietary fibre as non-starch polysaccharides with gas–liquid chromatographic, high-performance liquid chromatographic or spectrophotometric measurement of constituent sugars. Analyst.

[20] Kirk TK, Obst JR (1988). Lignin determination. Methods Enzymol.

[21] Choi S, Ray HE, Lai S-H, Alwood JS, Globus RK (2016). Preservation of multiple mammalian tissues to maximize science return from ground based and spaceflight experiments. PLoS One.

[22] Vandesompele J, De Preter K, Pattyn F, Poppe B, Van Roy N, De Paepe A, Speleman F. Accurate normalization of real-time quantitative RT-PCR data by geometric averaging of multiple internal control genes. Genome Biol. 2002;3:research0034. 1. 10.1186/gb-2002-3-7-research0034 PMC126239 12184808

[23] Hellemans J, Mortier G, De Paepe A, Speleman F, Vandesompele J. qBase relative quantification framework and software for management and automated analysis of real-time quantitative PCR data. Genome Biol 2007;8:R19. 10.1186/gb-2007-8-2-r19 PMC1852402 17291332

[24] Kheravii SK, Swick RA, Choct M, Wu S-B (2017). Dietary sugarcane bagasse and coarse particle size of corn are beneficial to performance and gizzard development in broilers fed normal and high sodium diets. Poult Sci.

[25] Sacranie A, Iji P, Choct M, Scott T. Reflux of digesta and its implications for nutrient digestion and bird health. Australian Poultry Science Symposium. 2005:171–5.

[26] Svihus B (2014). Function of the digestive system. J Appl Poult Res.

[27] Hetland H, Svihus B (2001). Effect of oat hulls on performance, gut capacity and feed passage time in broiler chickens. Br Poult Sci.

[28] Jiménez-Moreno E, Frikha M, de Coca-Sinova A, García J, Mateos GG (2013). Oat hulls and sugar beet pulp in diets for broilers 1. Effects on growth performance and nutrient digestibility. Anim Feed Sci Technol.

[29] Yokhana J, Parkinson G, Frankel T (2016). Effect of insoluble fiber supplementation applied at different ages on digestive organ weight and digestive enzymes of layer-strain poultry. Poult Sci.

[30] Bogułsawska-Tryk M (2005). Effect of different levels of cellulose in the diet on the proteolytic activity of the pancreas in broiler chickens. Folia Biol.

[31] Svihus B, Juvik E, Hetland H, Krogdahl Å (2004). Causes for improvement in nutritive value of broiler chicken diets with whole wheat instead of ground wheat. Br Poult Sci.

[32] Murai A, Satoh S, Okumura J-i, Furuse M (2000). Factors regulating amylase secretion from chicken pancreatic acini in vitro. Life Sci.

[33] Chandra R, Liddle RA (2009). Neural and hormonal regulation of pancreatic secretion. Curr Opin Gastroenterol.

[34] Kheravii SK, Swick RA, Choct M, Wu S-B. Roles of sugarcane bagasse and corn particle size in nutrient digestibility and gizzard in broilers. European symposium on poultry. Nutrition. 2017;

[35] Li P, Yin Y-L, Li D, Kim SW, Amino WG (2007). Acids and immune function. Br J Nutr.

[36] Hatzoglou M, Fernandez J, Yaman I, Closs E (2004). Regulation of cationic amino acid transport: the story of the CAT-1 transporter. Annu Rev Nutr.

[37] Duke G (1982). Gastrointestinal motility and its regulation. Poult Sci.

[38] Karasawa Y (1999). Significant role of the nitrogen recycling system through the ceca occurs in protein-depleted chickens. J Exp Zool.

[39] Sacranie A. How feed constituents regulate gut motility, feed utilisation and growth in broiler chickens. Doctor of Philosophy thesis: University of New England. 2010.

[40] Gilbert E, Li H, Emmerson D, Webb K, Wong E (2007). Developmental regulation of nutrient transporter and enzyme mRNA abundance in the small intestine of broilers. Poult Sci.

[41] Paris N, Wong E (2013). Expression of digestive enzymes and nutrient transporters in the intestine of Eimeria maxima-infected chickens. Poult Sci.

[42] Ohkubo T, Shamoto K, Ogino T (2007). Structure and tissue distribution of cholecystokinin-1 receptor in chicken. J Poult Sci.

[43] Su S, Miska K, Fetterer R, Jenkins M, Wong E (2014). Expression of digestive enzymes and nutrient transporters in Eimeria acervulina-challenged layers and broilers. Poult Sci.

[44] Guo S, Liu D, Zhao X, Li C, Guo Y (2014). Xylanase supplementation of a wheat-based diet improved nutrient digestion and mRNA expression of intestinal nutrient transporters in broiler chickens infected with Clostridium perfringens. Poult Sci.

[45] Yang F, Lei X, Rodriguez-Palacios A, Tang C, Yue H (2013). Selection of reference genes for quantitative real-time PCR analysis in chicken embryo fibroblasts infected with avian leukosis virus subgroup J. BMC Res Notes.

[46] Li YP, Bang DD, Handberg KJ, Jorgensen PH, Zhang MF (2005). Evaluation of the suitability of six host genes as internal control in real-time RT-PCR assays in chicken embryo cell cultures infected with infectious bursal disease virus. Vet Microbiol.

